# Microwave-Assisted Method for Simultaneous Extraction and Hydrolysis for Determination of Flavonol Glycosides in *Ginkgo* Foliage Using Brönsted Acidic Ionic-Liquid [HO_3_S(CH_2_)_4_mim]HSO_4_ Aqueous Solutions

**DOI:** 10.3390/ijms13078775

**Published:** 2012-07-16

**Authors:** Huanhuan Yao, Xinxuan Du, Lei Yang, Wenjie Wang, Fengjian Yang, Chunjian Zhao, Xiangdong Meng, Lin Zhang, Yuangang Zu

**Affiliations:** State Engineering Laboratory for Bio-Resource Eco-Utilization, Northeast Forestry University, Harbin 150040, China; E-Mails: avfdvf@163.com (H.Y.); duxinxuan1985@163.com (X.D.); wjwang225@hotmail.com (W.W.); yangfj@nefu.edu.cn (F.Y.); zcjsj@163.com (C.Z.); mxdhrb@gmail.com (X.M.); zhanglin6600@sina.com (L.Z.)

**Keywords:** *Ginkgo biloba*, ionic liquid, [HO_3_S(CH_2_)_4_mim]HSO_4_, microwave-assisted extraction, hydrolysis, flavonol glycoside

## Abstract

The Brönsted acidic ionic-liquid [HO_3_S(CH_2_)_4_mim] HSO_4_, a novel dual catalyst–solvent, has been successfully applied in simultaneous microwave-assisted extraction and hydrolysis for the determination of flavonol glycosides in *Ginkgo* foliage. The parameters, namely the [HO_3_S(CH_2_)_4_mim]HSO_4_ concentration, microwave-irradiation power, microwave-irradiation time, and solid–liquid ratio, were optimized. The optimum conditions were: an amount of 1.5 M [HO_3_S(CH_2_)_4_mim]HSO_4_, a microwave-irradiation power of 120 W, an irradiation time of 15 min, and a solid–liquid ratio of 1:30 g/mL. Compared with traditional methods the proposed approach demonstrates higher efficiency in a shorter operating time, and is an efficient, rapid, and simple sample preparation method.

## 1. Introduction

*Ginkgo* foliage has been one of the most popular traditional Chinese medicines for several thousand years [1]. Today, it is one of the top-selling herbs in the United States and Europe. Extracts of *Ginkgo biloba* (Egb) have been proven to be clinically effective in the treatments of peripheral vascular diseases, Alzheimer’s disease, dementia in elderly people, and tinnitus. The extracts have a number of functions. For example, they inhibit platelet aggregation [2], act as antioxidants and radical scavengers, and protect the central nervous system [3,4]. The primary active components are flavonol glycosides (24%) and a small amount of terpene lactones (6%) [5]. The flavonoids are primarily quercetin, kaempferol, and isorhamnetin. There are Egb products, including drugs and health foods, on the market in many countries.

There are many papers in the literature on the analysis and extraction techniques for *Ginkgo* flavonol glycosides. Traditionally, the extraction and hydrolysis of flavonol glycosides were separate procedures. The flavonol glycosides of *Ginkgo* were isolated by Soxhlet, maceration and heat reflux extractions [6,7] with volatile organic solvents, including methanol, ethanol, acetone and some mixed solvents. In contrast, the hydrolysis of the various Egb glycosides to their three primary aglycones is commonly achieved by refluxing the sample with aqueous/ethanolic hydrochloric acid for approximately 2 h. These conventional extraction techniques are inconvenient, inefficient, time-consuming, energy-consuming, result in degradation of target components, involve large volumes of toxic organic solvents and lead to unsatisfactory recoveries. It is therefore necessary to develop new and effective methods to find alternatives to traditional techniques.

Recently, microwave-assisted extraction has been accepted as a potential and powerful alternative to conventional techniques for the extraction of target components of plant materials. There have been many studies showing that microwave-assisted extraction is more effective than conventional methods for the extraction of flavonol glycosides from medicinal plants [8,9]. The extraction time was reduced, less solvent was used, and increased amounts of flavonol glycosides were extracted. Microwave hydrolysis has already been successfully used in food sample analysis, for example, for the determination of amino acids, choline, and vitamin B_12_ [10,11].

Ionic liquids are organic salts that exist as liquids below a threshold temperature. They are composed of large organic cations and small inorganic or organic anions [12]. They have recently attracted much research interest for a variety of applications because of their excellent properties: extremely low vapor pressure, high thermal stability, wide liquid range, tunable viscosity, miscibility with water and organic solvents, and good solubility and extractability for various organic compounds; moreover, ionic liquids can efficiently absorb and transfer microwave energy [13,14]. In the past few years, ionic liquids have been used as attractive green alternatives to conventional volatile organic solvents in various applications, including analytical applications [15–17], organic synthesis, catalysis [13,18,19] and separation [20–23]. Ionic liquids have also been widely used as catalysts in many reactions, such as alkylation, esterification, Michael addition, oligomerization and rearrangement. However, there have been few studies of hydrolysis reactions using ionic liquids as catalysts. This might be because one of the limitations of the traditional ionic liquids, namely the very weak acidities [24], makes it hard to obtain good catalytic activities in hydrolysis reactions using ionic liquids as catalysts. Although some studies have investigated the effects of ionic liquids in hydrolysis reactions, the ionic liquids, for example 1-butyl-3-methylimidazolium chloride, 1-butyl-3-methylimidazolium bromide ([Bmim]Br), and 1-butyl-3-methylimidazolium tetrafluoroborate, were only used as solvents or additives to enhance enzymatic or acid-catalyzed processes [25,26]. Recently, the introduction of Brönsted acidic functional groups into the cations or anions of ionic liquids, especially SO_3_H functional groups, which obviously enhance their acidities [27–29], has shown great promise for the use of ionic liquids as green catalysts with good catalytic activities in hydrolysis reactions. [HO_3_S(CH_2_)_4_mim]HSO_4_ is a Brönsted acidic ionic liquid that can be used to extract flavonol glycosides and to catalyze their hydrolysis.

In the present study, the potential of the Brönsted acidic ionic liquid [HO_3_S(CH_2_)_4_mim]HSO_4_ as a dual catalyst-solvent in the microwave-assisted simultaneous extraction and hydrolysis of flavonol glycosides from *G. biloba* was investigated. Various parameters influencing the procedure were optimized systematically. The ionic-liquid-based microwave-assisted simultaneous extraction and hydrolysis (ILMASEH) approach developed here was compared with conventional approaches. The simultaneous extraction and hydrolysis of flavonol glycosides demonstrated that the proposed ILMASEH approach showed great potential for easy quality assessment of *G. biloba* as well as of other flavonoid-rich plants.

## 2. Results and Discussion

### 2.1. Effect of Solvent Concentration

**Figure 1:**
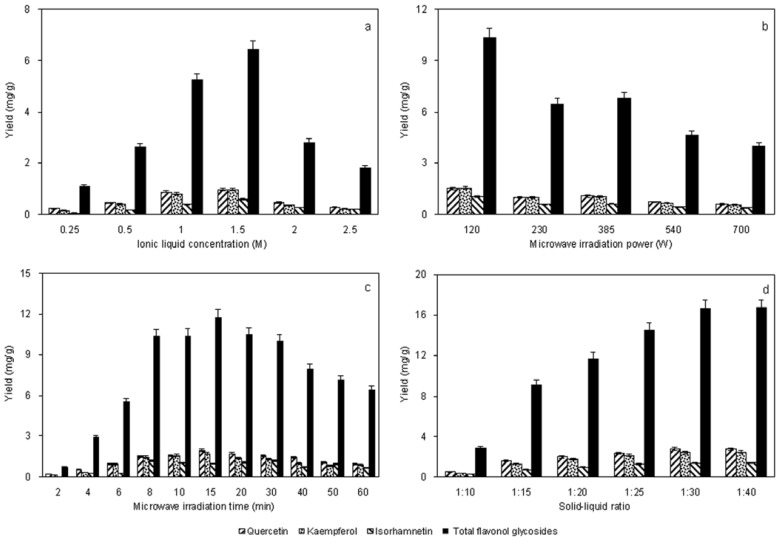
Effects of [HO_3_S(CH_2_)_4_mim]HSO_4_ concentration: (**a**) microwave power, (**b**) microwave time, (**c**) solid–liquid ratio and (**d**) ionic-liquid-based microwave-assisted simultaneous extraction and hydrolysis (ILMASEH) process was performed in a microwave unit with a power of 700 W. Dried sample was mixed with [HO_3_S(CH_2_)_4_mim]HSO_4_ in water at different concentrations and then irradiated with microwaves for specified times.

The optimum [HO_3_S(CH_2_)_4_mim]HSO_4_ concentration in aqueous solution for ILMASEH was determined by carrying out extractions and hydrolyses with [HO_3_S(CH_2_)_4_mim]HSO_4_ solutions of different concentrations (from 0.25 to 2.5 M). In the experiments, 1.0 g of sample was soaked in 30 mL of [HO_3_S(CH_2_)_4_mim]HSO_4_ and then extracted under a microwave-irradiation power of 120 W and an irradiation time of 15 min. The results in Figure 1a show that the yield increased in the [HO_3_S(CH_2_)_4_mim]HSO_4_ concentration range 0.25–1.5 M. Upon further increases, however, a slight decrease in the yield was observed. The 1.5 M [HO_3_S(CH_2_)_4_mim]HSO_4_ solution was therefore selected as the optimum ionic-liquid concentration. We speculate that the degradation or isomerization of aglycones often increases at high ionic liquid concentrations, which could lead to a decrease in the calculated yield of glycosides.

### 2.2. Effect of Microwave-Irradiation Power

Optimization of the microwave power used during ILMASEH is very important for ensuring efficient extraction and hydrolysis, and the effect of this variable was examined. In the experiments, 1.0 g of sample was soaked in 30 mL of 1.5 M [HO_3_S(CH_2_)_4_mim]HSO_4_ and then extracted under irradiation for 15 min. The yields obtained at different powers (120, 230, 385, 540 and 700 W) gradually decreased as the microwave power increased from 120 to 700 W. The ILMASEH time was maintained constant at 10 min throughout the experiments. It was found that as the microwave power increased, the yields decreased consistently (Figure 1b). This indicated that high-power microwave irradiation might result in carbonization of the raw materials as a result of internal overheating, isomerization of flavonol aglycones, and energy consumption. This phenomenon was also observed for ionic-liquid microwave-assisted extraction of lignans by Ma *et al*. [17]. The minimum microwave power tested (120 W) was therefore the optimum power for maximum yield.

### 2.3. Effect of Microwave-Irradiation Time

In the experiments, 1.0 g of sample was soaked in 30 mL of 1.5 M [HO_3_S(CH_2_)_4_mim] HSO_4_ and then extracted under a microwave-irradiation power of 120 W. The influence of the microwave-irradiation time on the yields of flavonol aglycones was examined over the range of 2–60 min, and the results are shown in Figure 1c. The results show that when the irradiation time was increased from 2 to 15 min, the yields of the three flavonol aglycones increased significantly. The yields of the three flavonol aglycones were low during the first 15 min indicating that more time was needed for microwave irradiation to disrupt the cell walls, aid the release of the flavonol glycosides into the solvent, and effectively hydrolyze the flavonol glycosides. On prolonged application of microwave irradiation, for more than 15 min, the yields of the three flavonol aglycones from *Ginkgo* foliage decreased, which indicated that the higher temperature probably caused degradation or isomerization of the flavonol aglycones; similar results were obtained in the extraction of alkaloids from *Camptotheca acuminate* [23] and of saponins from *Ganoderma atrum* [30]. The application of microwaves for 15 min was therefore selected for all subsequent experiments.

### 2.4. Effect of Solid–Liquid Ratio

The solid–liquid ratio is a crucial factor and was also studied to optimize the yields. Large solvent volumes could make the procedure difficult and lead to unnecessary waste, but small volumes may lead to incomplete extraction and hydrolysis. A series of experiments was carried out with different solid–liquid ratios (1:10, 1:15, 1:20, 1:25, 1:30 and 1:40 *w*/*v*) in 1.5 M [HO_3_S(CH_2_)_4_mim]HSO_4_ under a microwave-irradiation power of 120 W and an irradiation time of 15 min to evaluate the effect of the solid–liquid ratio. Figure 1d shows that the yields of the three flavonol aglycones increased with increasing solvent volume for solid–liquid ratios up to 1:30. Higher solvent volumes, however, did not significantly improve the yields of the flavonol aglycones. A solid–liquid ratio of 1:30 was therefore chosen as the optimum solid-liquid ratio.

Based on the above experiments, the optimum ILMASEH conditions were found to be as follows: 1.5 M [HO_3_S(CH_2_)_4_mim]HSO_4_ as the extraction solvent, a solid-liquid ratio of 1:30 (*w*/*v*), a microwave power of 120 W, and an irradiation time of 15 min.

### 2.5. Comparison of ILMASEH Approach with Conventional Methods

In the present study, pharmacopoeia of the People’s Republic of China sample preparation method (PPRCM), European pharmacopoeia sample preparation method (EPM), United States pharmacopeia and national formulary sample preparation method (USP-NFM), acidic ethanol microwave-assisted sample preparation method (AEMM), 1-butyl-3-methylimidazolium bromide ([Bmim]Br) microwave-assisted sample preparation method (BMM) and acidic [Bmim]Br microwave-assisted sample preparation method (ABMM) techniques were compared with respect to their yields of total flavonol glycosides from *Ginkgo* foliage. The total flavonol glycosides yields obtained using these six methods are summarized in Table 1. The total flavonol glycosides yields obtained using ILMASEH and ABMM were higher than those obtained using AEMM, PPRCM, EPM, and USP-NFM. In ILMASEH and ABMM, the flavonol glycosides in *Ginkgo* foliage could absorb sufficient microwave energy to be quickly transferred into the extraction solvent and hydrolyzed; the extraction time was significantly reduced and the yields increased considerably. This showed that compared with traditional methods, ILMASEH used only a small amount of ionic liquid and could obtain higher yields in shorter extraction times (15 min compared with 6.50 h for PPRCM, 2.75 h for EPM, and 2.25 h for USP-NFM), indicating that the [HO_3_S(CH_2_)_4_mim] HSO_4_ ionic-liquid was a dual catalyst–solvent and that ILMASEH was a more rapid and efficient method.

### 2.6. Method Validation

The stabilities of the aglycones under the experimentally derived optimum conditions were assessed by subjecting standards of quercetin, kaempferol, and isorhamnetin to microwave irradiation for 15 min at a microwave power of 120 W. The recoveries of the aglycones were assumed to be indicative of the stability of the aglycones under the ILMASEH conditions used (Table 2). The average complete recovery under the ILMASEH operating conditions varied from 98.0% to 99.3% with no changes in retention times observed for the aglycones. Degradation is therefore insignificant under the selected optimum conditions. For the standards in 1.5 M [HO_3_S(CH_2_)_4_mim]HSO_4_ solution stored for 7 days, the average recoveries of quercetin, kaempferol and isorhamnetin were 97.0%, 92.7% and 91.0%, respectively.

Under the optimized conditions detailed above, three samples of *Ginkgo* foliage, which had been spiked with quercetin, kaempferol and isorhamnetin were extracted and the recoveries were analyzed; the results are shown in Table 3. The recoveries of quercetin, kaempferol, and isorhamnetin from *Ginkgo* foliage were 99.49%, 99.75% and 100.29%, respectively.

To determine the repeatability of the novel method, five samples of the same weight (0.5 g) were processed under the optimum conditions. The mean yields of quercetin, kaempferol and isorhamnetin obtained under the optimized conditions showed good repeatability with calculated RSD values of 3.3%, 4.2% and 3.9%, respectively. This shows that the proposed ILMASEH method has an acceptable level of repeatability.

The results suggested that quercetin, kaempferol and isorhamnetin were stable in the ionic-liquid solutions and in the extracts. These method validation studies indicate that the proposed method is credible.

## 3. Experimental Section

### 3.1. Materials and Chemicals

*Ginkgo* foliage was purchased from the Tancheng Medicinal Materials Market (Shandong, China). The dried samples were powdered to a homogeneous size and then sieved (60–80 mesh) prior to use. Quercetin, kaempferol, and isorhamnetin standards were purchased from the National Institute for the Control of Pharmaceutical and Biological Products (Beijing, China). HPLC-grade methanol (J&K Chemical Ltd. Beijing, China) and ultrapure water prepared using a Milli-Q purification system (Millipore, Bedford, MA, USA) were used as the HPLC mobile phase. Ionic liquids, [HO_3_S(CH_2_)_4_mim]HSO_4_ and [Bmim]Br, were obtained from Chengjie Co., Ltd. (Shanghai, China) and used without further purification. Some physicochemical properties of the ionic liquids studied are listed in Table 4. All other chemicals used in this study were of at least analytical grade and were obtained from Sinopharm Chemical Reagent Co., Ltd (Shanghai, China). All solutions and samples prepared for chromatographic analysis were filtered through a 0.45-μm microporous membrane (Guangfu, Tianjin, China) before being injected onto the HPLC column.

### 3.2. HPLC Analysis and Quantification

The HPLC system consisted of a Waters 717 autosampler, a 1525 binary pump, a 717 automatic column temperature control compartment and a 2487 UV-detector (Waters, Milford, MA, USA). Chromatographic separation was performed on a Kromasil 100-5C_18_ reversed-phase column (4.6 mm × 250 mm, 5 μm, AkzoNobel, Bohus, Sweden).

Stock solutions of quercetin, kaempferol, and isorhamnetin were prepared in methanol at a concentration of 30 μg/mL. Working standard solutions were prepared by serial dilution of the stock solutions in methanol and then stored at 1–4 °C in darkness until the HPLC analysis.

**Figure 2:**
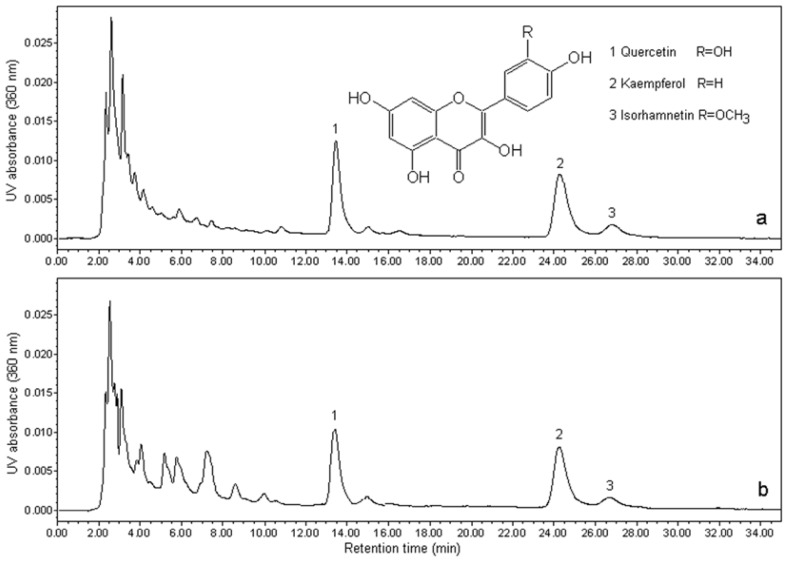
Comparative HPLC chromatograms for quercetin, kaempferol, and isorhamnetin in an extract obtained using ILMASEH (1.5 M [HO_3_S(CH_2_)_4_mim]HSO_4_ as dual catalyst–solvent) (**a**) and using EPM method (**b**). Inset: chemical structures of three predominant flavonol aglycones in *Ginkgo biloba*.

The extracts were directly injected into the liquid chromatograph. The mobile phase was composed of methanol: 1% aqueous acetic acid solution (52.5:47.5, *v*/*v*). The flow rate was 1 mL/min and the injection volume was 10 μL. The column temperature was maintained at 25 °C. The UV detection wavelength was 360 nm, where quercetin, kaempferol, and isorhamnetin have optimum absorbances. Under these conditions, the quercetin, kaempferol and isorhamnetin were separated sufficiently (Figure 2). Quercetin, kaempferol, and isorhamnetin were identified by comparing their retention times with the corresponding peaks in the standard solutions.

The corresponding calibration curves for each compound are *Y*_quercetin_ = 258971*x* − 34818 (*r* = 0.9991), *Y*_kaempferol_ = 254792*x* + 8635.7 (*r* = 0.9993), and *Y*_isorhamnetin_ = 100039*x* + 34462 (*r* = 0.9995). Good linearities were found for quercetin, kaempferol, and isorhamnetin in the range of 2.5–30 μg/mL.

### 3.3. Ionic Liquid-based Microwave-Assisted Simultaneous Extraction and Hydrolysis (ILMASEH)

**Figure 3:**
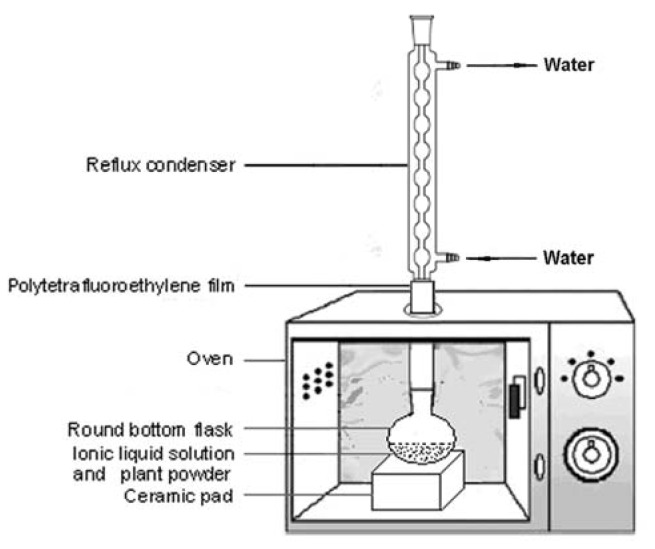
Schematic diagram of ionic liquids based microwave-assisted simultaneous extraction and hydrolysis apparatus.

The experimental setup is shown schematically in Figure 3. A household microwave oven (Glanz, Shunde, China) was used for ILMASEH with at a microwave irradiation frequency of 2450 MHz. The maximum output power of the oven was 700 W. In order to examine the effect of the microwave power, five power levels, 100% (700 W), 77% (540 W), 55% (385 W), 33% (230 W), and 17% (120 W) were studied. The whole system was run at atmospheric pressure.

The dimensions of the interior cavity of the oven were 215 × 350 × 330 mm^3^. The microwave oven was modified by drilling a hole in the top. A round-bottomed flask of a capacity of 50 mL was placed in the oven and connected to a reflux condenser through the hole. After placing the reflux condenser in the oven, the hole around the neck of the flask was covered with polytetrafluoroethylene (PTFE) to prevent microwave leakage.

A dried sample (0.5 g) was mixed with 15 mL of ionic-liquid solution in a 50 mL flask and then the suspension was irradiated with microwaves. The optimum concentration of the selected ionic liquid, microwave power, irradiation time, and solid–liquid ratio was systematically studied in this work. The extracts obtained were rapidly cooled to room temperature using a cold bath and filtered through a 0.45-μm PTFE microporous membrane (Guangfu, Tianjin, China) for subsequent HPLC analysis.

The flavonol glycoside concentrations were determined from the peak areas of quercetin, kaempferol and isorhamnetin using the following equation:

(1) $$\text{Individual glycoside }(\text{mg}/\text{g})=(C\times F_{V}\times F)/(W\times 1000)$$

where *C* is the aglycone concentration determined from the standard curve, *F_V_* is the final volume (mL), *F* is a conversion factor (2.504 for quercetin, 2.588 for kaempferol, and 2.437 for isorhamnetin) [31], and *W* is the sample weight (g). So,

(2) $$\begin{array}{l} \text{Total flavonol}\;\text{glycosides }(\text{mg}/\text{g})=\text{quercetin glycosides }(\text{mg}/\text{g})+\\ \text{kaempferol glycosides}\;(\text{mg}/\text{g})+\text{isorhamnetin glycosides }(\text{mg}/\text{g}) \end{array}$$

### 3.4. Pharmacopoeia of the People’s Republic of China Sample Preparation Method (PPRCM)

Powdered *Ginkgo* foliage was weighed (1.0 g) and transferred to a Soxhlet extractor. Chloroform (100 mL) was added and the mixture was heated under reflux for 2 h. The chloroform solution was discarded, 100 mL of methanol was added, and the mixture was heated under reflux for 4 h. The liquid extract was decanted and concentrated under a vacuum to dryness. A methanol/25% HCl (4:1) solution (25 mL) was added and the mixture was refluxed for 0.5 h [32]. An aliquot of the sample was filtered (PTFE, 0.45 μm, Guangfu, Tianjin China) prior to HPLC analysis.

### 3.5. European Pharmacopoeia Sample Preparation Method (EPM)

Powdered *Ginkgo* foliage was weighed (2.5 g) and transferred to a 250 mL boiling flask. Acetone solution (60% *v*/*v*, 50 mL) was added and the mixture was heated under reflux for 70 min. The liquid extract was decanted and filtered, and 40 mL of 60% *v*/*v* acetone solution were added to the remaining solid. The mixture was heated under reflux for an additional 70 min. The sample was cooled and filtered into a 100 mL volumetric flask containing the initial extract. The final extract (50 mL) was transferred to a 250 mL round-bottomed flask. The contents were evaporated under a vacuum until the steady flow of condensed solvent ceased. The contents were transferred to a 50-mL volumetric flask, 4.4 mL of hydrochloric acid were added, and the sample was diluted to volume with methanol. The flask contents were centrifuged for approximately 5 min. Aliquots (10 mL) of supernatant were transferred to 10-mL glass vials and the vials were sealed with aluminum caps. The vials were submerged in a boiling-water bath for 25 min [33]. An aliquot of the sample was filtered (PTFE, 0.45 μm, Guangfu, Tianjin, China) prior to HPLC analysis.

### 3.6. United States Pharmacopeia and National Formulary Sample Preparation Method (USP-NFM)

Powdered *Ginkgo* foliage was weighed (1.0 g) and transferred to a 250 mL boiling flask. Ethanol (50 mL), water (20 mL), and hydrochloric acid (8 mL) were added and the mixture was refluxed for approximately 135 min. The sample was cooled to room temperature and filtered into a 100 mL volumetric flask. The sample was diluted to volume with water and mixed well by inversion [34]. An aliquot of the sample was filtered (PTFE, 0.45 μm, Guangfu, Tianjin, China) prior to HPLC analysis.

### 3.7. Other Reference Sample Preparation Methods

Acidic ethanol microwave-assisted sample preparation method (AEMM): Powdered *Ginkgo* foliage was weighed (1.0 g) and transferred to a 250 mL boiling flask. Ethanol (50 mL), water (20 mL), and hydrochloric acid (8 mL) were added, and the mixture was subjected to microwave irradiation for 15 min at 120 W. The subsequent steps were the same as those in USP-NFM.

[Bmim] Br microwave-assisted sample preparation method (BMM): [Bmim] Br was used as the solvent in the sample preparation. The experiments were operated under the optimum conditions, except for the solvent type. The subsequent steps were the same as those in ILMASEH.

Acidic [Bmim] Br microwave-assisted sample preparation method (ABMM): A 1.5 M [Bmim] Br acidic solution (pH 0.5, adjusted with 6.0 mol/L of hydrochloric acid) was used as the solvent in the sample preparation. The experiments were operated under the optimum conditions, except for the solvent type. The subsequent steps were the same as those in ILMASEH.

## 4. Conclusions

The application of ILMASEH was successfully developed for simultaneous extraction and hydrolysis for the determination of flavonol glycosides in *Ginkgo* foliage. The ILMASEH conditions, namely the [HO_3_S(CH_2_)_4_mim]HSO_4_ concentration, microwave-irradiation time, microwave-irradiation power and solid-liquid ratio, were optimized. The highest yield of total flavonol glycosides was obtained using 0.5 g of sample powder mixed with 15 mL of 1.5 M [HO_3_S(CH_2_)_4_mim]HSO_4_ and subjecting this to microwave irradiation for 15 min at a microwave power of 120 W, the yield of total flavonol glycosides was the highest. The proposed ILMASEH approach is an efficient, rapid and simple sample preparation method.

## Figures and Tables

**Table 1:** Comparison of ILMASEH approach with reference and conventional methods.

Methods	Solvents	Solvent volume (mL/g)	Extraction method	Power (W)	Heating time (h)	Yield (mg/g)			

						Quercetin	Kaempferol	Isorhamnetin	Total flavonol glycosides
ILMASEH	1.5 M [HO_3_S(CH_2_)_4_mim]HSO_4_	35	Microwave	120	0.25	2.75 ± 0.13	2.36 ± 0.11	1.37 ± 0.06	16.33 ± 0.76
PPRCM	Chloroform/Methanol/Hydrochloric acid	225	Soxhlet	500	6.50	2.45 ± 0.12	1.90 ± 0.08	1.19 ± 0.06	13.94 ± 0.65
EPM	Acetone-H_2_O (60:40) Hydrochloric acid	Approx. 60	Reflux	500	2.75	2.35 ± 0.14	1.95 ± 0.11	1.23 ± 0.07	13.93 ± 0.81
USP-NFM	Ethanol-H_2_O-Hydrochloric acid (50:20:8)	78	Reflux	500	2.25	2.44 ± 0.11	2.19 ± 0.12	1.35 ± 0.07	15.07 ± 0.76
AEMM	Ethanol-H_2_O-Hydrochloric acid (50:20:8)	78	Microwave	120	0.25	2.56 ± 0.12	2.15 ± 0.10	1.20 ± 0.06	14.83 ± 0.71
BMM	1.5 M [Bmim]Br	78	Microwave	120	0.25	0.57 ± 0.02	0.69 ± 0.03	0.30 ± 0.01	3.92 ± 0.15
ABMM	1.5 M [Bmim]Br (pH 0.5, adjusted with HCl)	78	Microwave	120	0.25	2.71 ± 0.14	2.45 ± 0.11	1.35 ± 0.06	16.34 ± 0.78

**Table 2:** Stability studies of quercetin, kaempferol, and isorhamnetin standards under optimum ILMASEH conditions. ILMASEH conditions: microwave power 120 W, microwave time 15 min, 1:30 solid-liquid ratio, prepared with 1.5 M [HO_3_S(CH_2_)_4_mim]HSO_4_.

Compounds	Initial concentration (mg mL^−1^)	Recovered concentration after ILMASEH (mg mL^−1^)	RSD% (*n* = 3)	Average recovery (%)	Recovered concentration after 7 day (mg mL^−1^)	RSD% (*n* = 3)	Average recovery (%)
Quercetin	2.00	1.97	0.96	98.5	1.94	0.97	97.0
Kaempferol	1.50	1.48	0.97	99.3	1.39	0.99	92.7
Isorhamnetin	1.00	0.98	1.01	98.0	0.91	1.03	91.0

**Table 3:** Recovery of quercetin, kaempferol and isorhamnetin from *Ginkgo* foliage (*n* = 3).

	Aglycone content of the sample (mg)			Amount of added aglycone standard (mg)			Amount of the sample determined with added aglycone standard (mg)			Recovery (%)		
				
Sample	Quercetin	Kaempferol	Isorhamnetin	Quercetin	Kaempferol	Isorhamnetin	Quercetin	Kaempferol	Isorhamnetin	Quercetin	Kaempferol	Isorhamnetin
1	2.80	2.46	1.39	1.40	1.30	0.50	4.23	3.72	1.91	100.71	98.94	101.06
2	2.80	2.46	1.39	2.80	2.60	1.00	5.57	5.1	2.41	99.46	100.79	100.84
3	2.80	2.46	1.39	4.20	3.90	1.50	6.88	6.33	2.86	98.29	99.53	98.96
Average										99.49	99.75	100.29

**Table 4:** Physicochemical properties of the ionic liquids studied.

Ionic liquid	Cation	Anion	Form (25 °C)	Solubility in H_2_O (g/100 mL)
[HO_3_S(CH_2_)_4_mim] HSO_4_	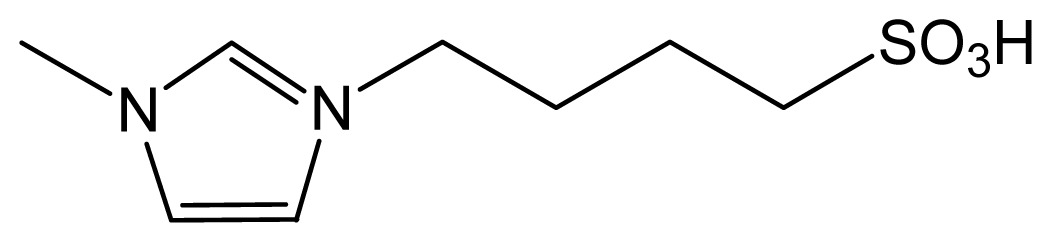	HSO_4_^−^	Liquid	Totally miscible
[Bmim] Br	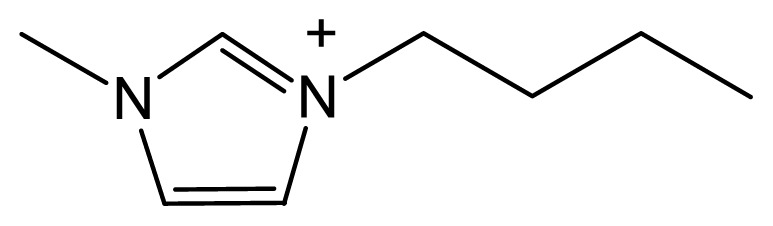	Br^−^	Solid	Totally miscible
